# Clinical and Para clinical Manifestations of Tuberous Sclerosis: A Cross Sectional Study on 81 Pediatric Patients

**Published:** 2012

**Authors:** Seyyed Hassan TONEKABONI, Parviz Tousi, Ahmad Ebrahimi, Farzad Ahmadabadi, Zarrintaj Keyhanidoust, Gholamreza Zamani, Morteza Rezvani, Susan Amirsalari, Azita Tavassoli, Alireza Rounagh, Alireza Rezayi

**Affiliations:** 1 Associate Professor of Pediatric Neurology, Pediatric Neurology Research Center, Shahid Beheshti University of Medical Sciences (SBMU), Tehran, Iran; 2 Professor of Dermatology, Skin Research Center, Shahid Beheshti University of Medical Sciences, Tehran, Iran; 3 Assistant Professor of Molecular Genetic, Shiraz University of Medical Sciences, Shiraz, Iran; 4 Fellowship of Pediatric Neurology, Assistant Professor of Pediatrics, Pediatric Neurology Research Center, Shahid Beheshti University of Medical Sciences (SBMU), Tehran, Iran; 5 Associate Professor of Pediatric Neurology, Tehran University of Medical Sciences, Tehran, Iran; 6 Assistant Professor of Pediatric Neurology, Children’s Medical Center, Tehran University of Medical Sciences (TUMS ),Tehran, Iran; 7 Assistan Professor of Pediatric Neurology, Qum University of Medial Sciences, Qum, Iran; 8 Associate Professor, Department of Pediatric Neurology, Faculty of Medicine, Baqiyatallah University of Medical Sciences, Tehran, Iran; 9 Assistant Professor of Pediatric Neurology, Tehran University of Medical Sciences, Tehran, Iran; 10 Assistant Professor of Pediatric Neurology, Alborz University of Medical Sciences, Karaj, Iran; 11 Fellowship of Pediatric Neurology, Pediatric Neurology Research Center, Shahid Beheshti University of Medical Sciences (SBMU), Tehran, Iran

**Keywords:** Tuberous sclerosis, Pediatrics, Epilepsy, Neurologic manifestations

## Abstract

**Objective:**

Tuberous sclerosis complex is an autosomal dominant neurocutaneous disease that presents with dermatological, neurological, cardiac, renal and ocular symptoms.

We described the variable clinical manifestations, neuroimaging findings, Age and sex distribution of tuberous sclerosis in a group of 81 patients referred to our clinic.

**Materials & Methods:**

Based on the diagnostic criteria, totally 81 tuberous sclerosis patients with sufficient data were enrolled into the study. These children were referred by child neurologists.

**Results:**

The mean age of the patients was 52 months (range, 7-180 months). There were 28 girls and 53 boys. A positive familial history of TSC was seen in 29.6% of the patients. Hypo pigmented macules were the most common manifestation (82.7%). Facial angiofibroma, shagreen patches, café-au-lait lesions and seizure were observed in 32.1%, 12.3%, 7.4%. and 74.1% of the studied cases, respectively. Infantile spasm was present in the clinical course of 32.1 % of the patients. Cortical tubers were the most common MRI finding which were seen in 21 cases (25.9%). Subepandymal giant cell astrocytoma was seen in four (4.9%) patients and intracranial calcification (detected by CT scan) was observed in 18 (22.2%) of the patients.

**Conclusion:**

Dermatological and neurological findings were the most common symptoms in tuberous sclerosis with a significant correlation between them. Thus, careful skin examination is necessary in epileptic patients for detection of the mentioned lesions.

## Introduction

Tuberous sclerosis complex (TSC) is an autosomal dominant neurocutaneous disease (phacomatosis) with variable clinical manifestations (1). The incidence of the disease is approximately 1/6000- 1/10000 (2, 3).

Diagnosis is based on clinical and paraclinical criteria defined by the tuberous sclerosis consensus conference in 1998 .There are two groups of symptoms including major and minor criterias.The major criterias consist of:Facial angiofibromas or forehead plaques, Nontraumatic ungula or periungual fibroma, Hypopigmented macules (more than 3), Shagreen patch, Cortical tubers, Subepandymal nodules, Subepandymal giant cell astrocytoma, Multiple retinal nodular hamartomas, Cardiac rhabdomyoma, Lymphangiomyomatosis and renal angiomyolipoma.The minor criterias include:Dental Pits (more than 14),Hamartomatous rectal polyps, Bone cysts, Cerebral whte matter radial migration lines, Nonrenal hamartomas, Retinal achromatic patch, Confetti skinlesions, Multiple renal cysts.When there are two major criteria or one major and two minor criteria the diagnosis is established as definite TSC. The term probable TS is used when one major and one minor criteria are detected (2). Only one major feature or two or more minor criteria without any major feature mentions the possibility of tuberous sclerosis (2).

The central nervous system involvement is the most common finding in tuberous sclerosis that leads to morbidity and mortality (4). These manifestations were first described by D M Bourneville in 1880. Before that, Von Recklinghausen had described a newborn who had died of respiratory distress and at post mortem examinations a great number of cerebral sclerosis was detected (3).

Epilepsy is the most common presenting symptom in tuberous sclerosis. In 98% of these patients seizure is discovered and 75% of them have a seizure attack in the first year of life (4). Seizure control is difficult and they tend to be refractory and intractable in more than 50% of the cases despite pharmacological and surgical treatment (5). Even cases with a good response to antiepileptic drugs in the beginning of therapy will have frequent relapses (6).

Surgery is one of the treatment options of seizures, but the outcome is best when the EEG-MRI and semiology of seizure indicate a surgically accessible location (6).

Recent reports on the direct approach to neoplastic lesions show that in cases with an increased intracranial pressure, surgical interventions may provide a better outcome (7). Surgery is not indicated in the presence of bilateral epileptogenic zones, progressive epileptic encephalopathy and severe mental retardation. In some cases with drug resistant epilepsy and those who are waiting for surgical intervention, the ketogenic diet has been considered. This diet can decrease more than 50%of seizures in 90% of cases (8). It seems that antiepileptic treatment before the onset of seizure reduces the severity of epilepsy and the risk of mental retardation in TSC if EEG shows multifocal activity without clinical seizure (9).

**Fig 1:**
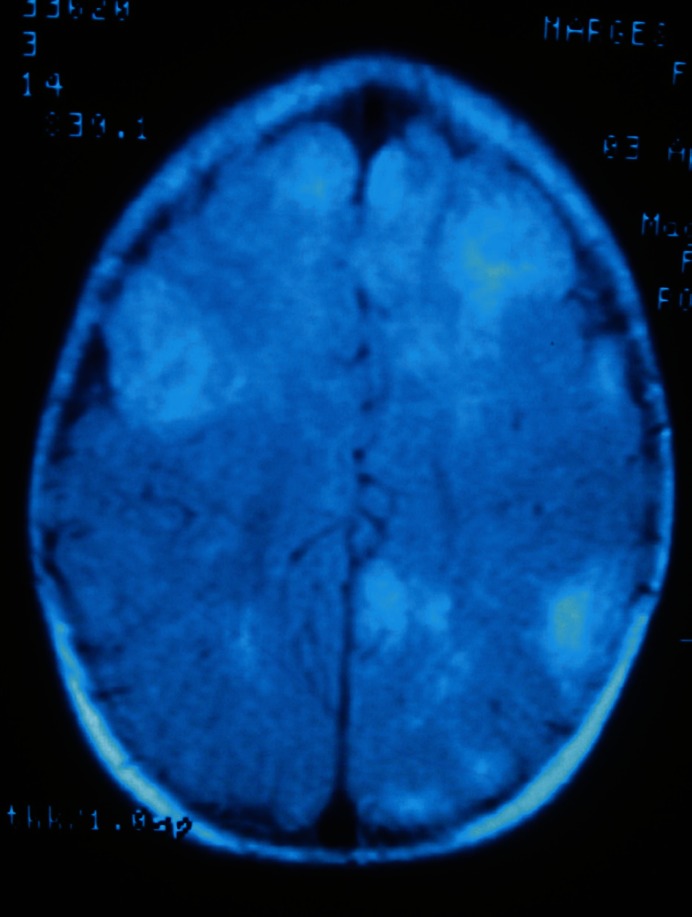
Cortical tubers

**Fig 2:**
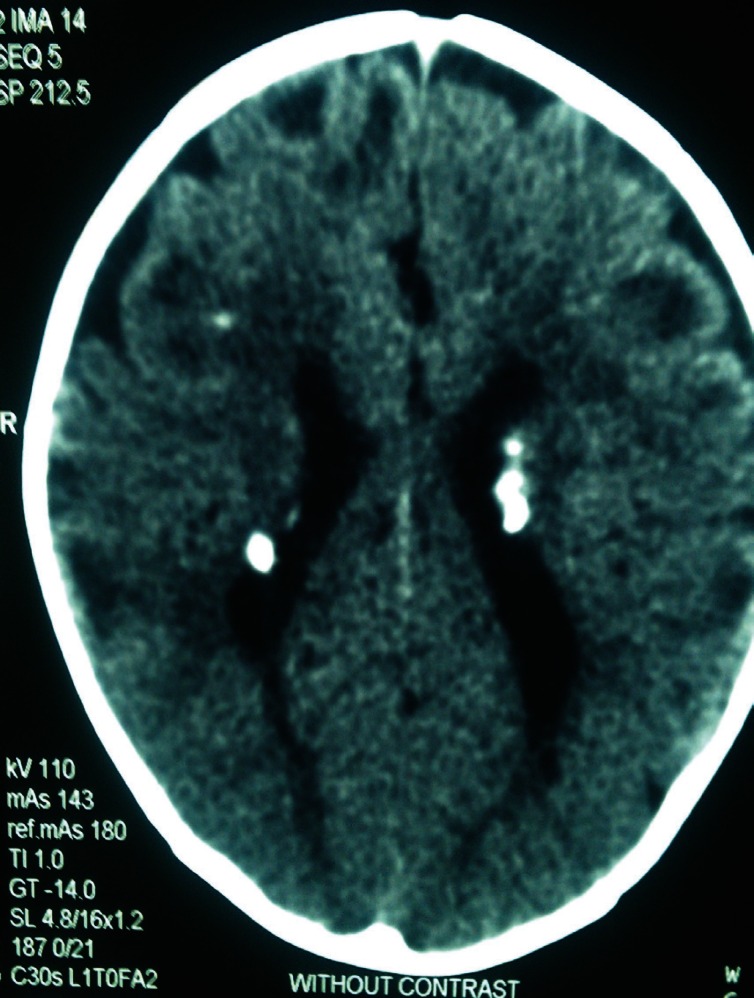
Subepandymal nodules & Cortical tubers

Other neurological manifestations of TSC include cortical tubers (Fig 1), subepandymal nodules (Fig 2) and subepandymal giant cell astrocytoma that may lead to seizure attacks. Cortical tubers tend to be in the front parietal region. Ventricular dilation is seen in 55% of TS cases and cerebral tumors in 1.7-15% with a mean age of 13.5 years (11). 

**Fig 3:**
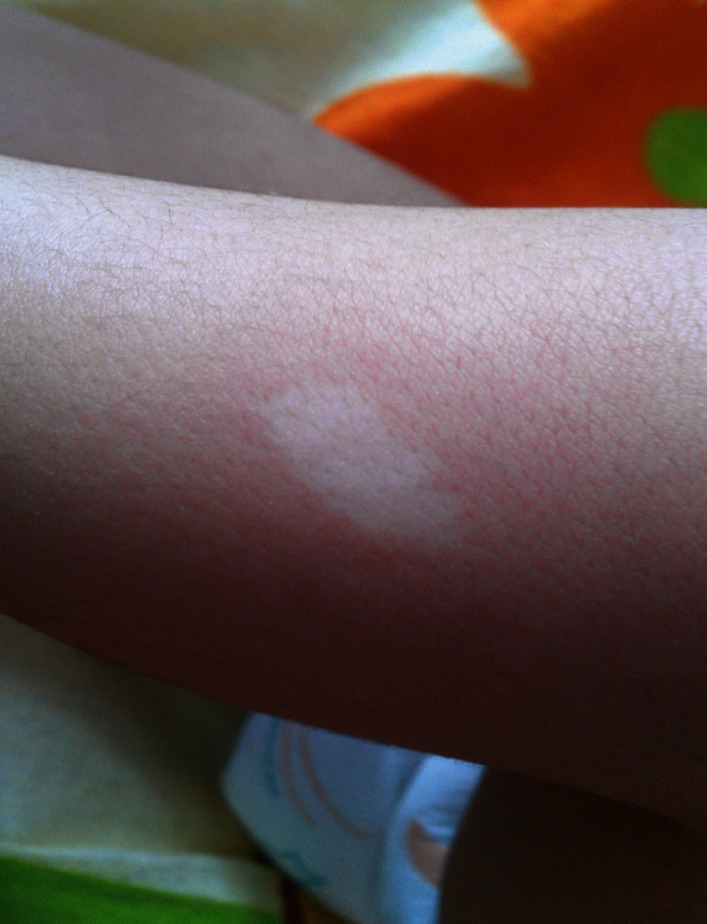
Ashleaf macules

The skin manifestations of TSC tend to be the most prevalent findings (4). The hypo pigmented macules or ash leaf lesions are the most important, early onset and characteristic dermatological findings (Fig 3). These lesions are seen in 4.7% of the normal population, but their prevalence is 97% in children with TSC (4). They are located on the trunk and buttocks and are easily identified with the wood lamp. Other skin lesions such as café-aulait macules, confetti like macules, periungual fibromas, forehead plaques and shagreen patches may also be observed. The latter is seen soon before puberty (3). 

Sometimes cardiac rhabdomyoma is the earliest diagnostic finding in TSC. These hamartomas of the heart remain asymptomatic and regress in size and number till 6 years of age in 58-74% of the patients (4). 

Renal involvement is seen as angiomyolipomas (AML) or cysts in TSCs. They are the second cause of death in these patients and the first fatal cause in patients older than 30 years of age. AML is the most common major renal finding and is often bilateral. Only a fair correlation was found between age and renal involvement (12). Ocular manifestations of TSC including retinal hamartomas occur in less than 50% of the patients and are bilateral in one third of the cases. There is no correlation between age and ocular manifestations (2).

## Materials & Methods

Eighty-one children (age range: 7 months to 15 years) based on the diagnostic criteria and sufficient data were enrolled into the study. These children were referred by dermatologists and child neurologists practicing in Tehran and other cities to the neurology clinic of Mofid children hospital from September 2009 till July 2012. Clinical and paraclinical information based on careful physical examination and systematic review of the patients were collected by a fellow in child neurology. 

This information was collected on a questionnaire and statistically analyzed by SPSS 12 package. Informed consent for collecting and publishing patients’ information was obtained from the parents.

## Results

The mean age of the patients at diagnosis was 52 months (age range, 7 to 180 months), the median was 48 months and sex distribution was 28 (34.6%) female and 53 (65.4%) male. Eleven patients (13.6%) were under 1 years, 38 patients(46.9%) between 1 and 5 years, 24 cases (29.6%) between 5 and 10 years and eight (9.9%) were between 10 and 15 years. 

A positive familial history of TSC was observed in 24 patients (29.6%). Sixteen families had a consanguineous marriage (19.8%) which is a normal finding in the Iranian population. 

The most common manifestation of disease, hypo pigmented macules (ash-leaf) were seen in 67 of the patients (82.7%). Other dermatological manifestations were less common. Shagreen patches were detected in ten (12.3%), café-au-lait lesions in 7.4% and facial angiofibroma in3.2% of our study group. 

Seizure was found in 60 patients (74.1%) and 32.1% of our study group had infantile spasm in their clinical course. The mean age of onset for infantile spasm was 5 months. The most common MRI finding was cortical tubers that were observed in 21 cases (25.9%). 

Subepandymal giant cell astrocytoma was found in four (4.9%) and intracranial calcification (detected by CT scan) was detected in 18 patients (22.2%) (table 1).

**Table 1:** Clinical and Paraclinical Manifestations of Tuberous Sclerosis in Our Study Group

**Manifestation**		**Number**	**Percentage**
Ophthalmological Symptoms	Retinal hamartoma	1	1.2%
	Achromatic retinal patches	0	0%
	Retinal astrocytoma	1	1.2%
	Optic glioma	0	0%
Cardiological Symptoms	Wolf-Parkinson-White syndrome	0	0%
	Cardiac rhabdomyoma	18	22.2%
Renal Symptoms	Renal cysts	5	6.2%
	Tumors of the Kidney	3	3.7%
Dermatological Symptoms	Forehead plaques	2	2.5%
	Facial angiofibroma	26	32.1%
	Ashleaf macules	67	82.7%
	Shagreen patch	10	12.3%
	Café-au-lait	6	7.4%
	Subungual fibroma	5	6.2%
	Skin tag	0	0%
	Confetti skin lesion	6	7.4%
	Poliosis	0	0%
Imaging Findings	Hamartomatous lesions of the brain	3	3.7%
	Cortical tubers	21	25.9%
	Subepandymal giant cell astrocytoma	4	4.9%
	Intracranial calcification	18	22.2%
CNS Symptoms	Infantile spasm	26	32.1%
	Seizure	60	74.1%
	Mental retardation	13	6%
	Learning difficulties	9	11.1
Endocrine Symptoms	Precocious puberty	0	0%
	Hypothyroidism	1	1.2%

Cardiac rhabdomyoma was seen in 18 patients (22.2%). No other cardiologic involvement (such as Wolf -Parkinson -White syndrome) was seen in our study. Ophthalmological involvements were found in two patients (one retinal hamartoma and one retinal astrocytoma). Renal problems were seen in eight cases including five cases of renal cysts (6.2%) and three cases of kidney tumors (3.7%).

**Fig 4:**
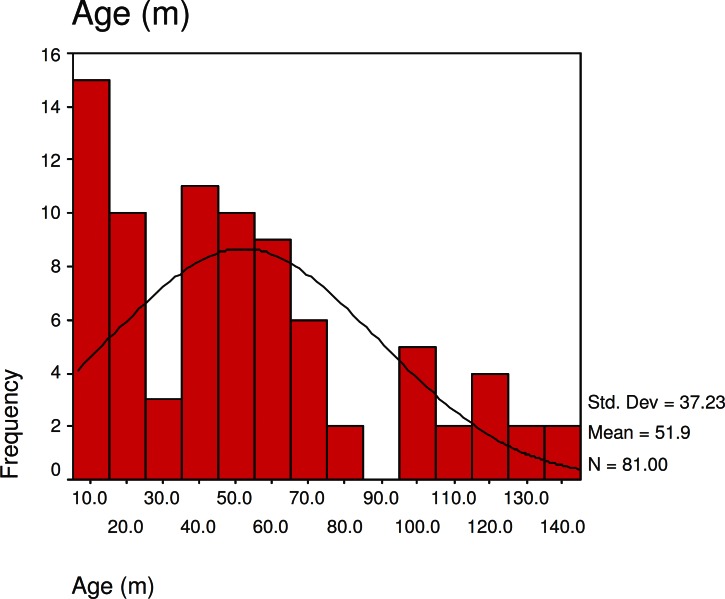
Age distribution of tuberous sclerosis patients in our study

## Discussion

The mean age of diagnosis in our study was 51 months and the median was 48 months. These parameters show a normal age distribution pattern. In prior studies, the mean age of diagnosis was higher than our study despite the lower median age (2). We think this difference is due to the age distribution of our study group. We performed our study in the pediatric group and other studies relied on all age groups so a different distribution was observed. Thirty percent of our patients (27 cases) had a positive familial history. In 11 cases, the mother and in 13 cases, the father were affected and in three cases, the siblings were affected without parental involvement. These show that despite the autosomal dominant pattern of tuberous sclerosis transmission, in many of the cases, it is the result of new mutations. In prior studies, the prevalence of sporadic tuberous sclerosis was 60-70% of the cases based on genetic (TSC1 or TSC2) (2, 13).

Only 1/5 of our patients were products of consanguineous marriage that is lower than the expected rate in the Iranian population (34-44%) (14). Hypo pigmented macules, the most common presenting sign of disease in our study, were seen in 82.7% of the cases. This sign was observed in 90-95% of the cases in prior studies. Considering the tendency of these lesions to disappear in adulthood, we expected more prevalence in our study due to the age distribution of the study group (2-4).

We found facial angiofibroma in 3.2% of the children. These lesions do not typically appear before the age of 3-4 years (2). In our study, the mean age of patients with angiofibroma was 46 years. Shagreen patches were observed in 12.3% of the patients. In other studies, the frequency of this finding was about 20-30% (15). These lesions are late onset lesions and usually appear soon before puberty. The mean age of shagreen patches in our patients was 76 months which is compatible with other studies.

Although lesions such as shagreen patches and facial angiofibroma are age dependent based on prior studies (2-4), we did not find any correlation between the age group and these lesions.

Other skin findings were less common probably due to the patient age distribution in our study group and they will probably show these manifestations at a later age in the course of their disease. 

Seizure was seen in 74% of our patients as the first presenting symptom. In prior studies, seizure was found in 47-60% of the patients, but the lifetime risk for seizure in tuberous sclerosis was approximately 84% (1, 2, 4, 15). The higher prevalence of seizure as the first presenting symptom in our study may be due to the fact that Mofid children hospital is a known child neurology referral center. So we are more confronted with neurologic manifestations in patients.

Infantile spasm was found in 32% of our patients, a range of 13%-15% was detected in other studies. The mean age of infantile spasm in our study was 5 months. There was a statistical correlation between the presentation of infantile spasm and the age of patients (P=0.05).

Cardiac rhabdomyoma was seen in 18 patients (22%). In prior studies its prevalence was about 12-30% (2, 4). It is the earliest finding in tuberous sclerosis in some studies (4). In eight cases of our study group, rhabdomyoma was found in the prenatal period. In other studies 30% of rhabdomyoma cases had been diagnosed in less than 6 months of age. It is compatible with our study (2). 

We found cortical tubers in 25.9% of the children. In comparison with other studies, it was less common probably due to the age distribution and severity of symptoms in our study. We did not find any cerebellar tubers. These lesions have been seen in 25% of TSC cases in other studies. So it is a late onset finding. There is a correlation between the severity of disease and the number of cortical tubers. In addition, cerebellar tubers are predictive for severe symptoms and more cognitive dysfunction (15). 

Intracranial calcification was observed in22.2% of our patients. They are most often found in the lateral ventricles and seem to occur more often anteriorly (15).

In 8 cases of our study group, rhabdomyoma was found in prenatal period. In other studies 30% of rhabdomyoma cases were diagnosed before6 month of age. (2, 4, 13).

Renal involvement was seen in 9% of our patients and renal cysts were the most common renal presentation of tuberous sclerosis similar to prior studies. There was no correlation between age-sex and renal involvement in our study.

We found a strong correlation between CNS involvement and skin manifestation in our study. This is also consistent with prior studies (15).
